# Pediatric Open Globe Injury in Central China

**DOI:** 10.3389/fmed.2021.762477

**Published:** 2022-01-24

**Authors:** Hongling Chen, Xianliang Zhang, Junjun Han, Xuemin Jin

**Affiliations:** ^1^Department of Ophthalmology, Henan Eye Institute, Henan Eye Hospital, Henan Provincial People's Hospital, People's Hospital of Zhengzhou University, Zhengzhou, China; ^2^Department of Ophthalmology, The First Affiliated Hospital of Zhengzhou University, Zhengzhou, China

**Keywords:** pediatric, Central China, open-globe injury, trauma, epidemiology

## Abstract

**Purpose:**

To describe the characteristics, managements, and outcomes of pediatric open globe injury (OGI) in central China.

**Methods:**

Retrospective chart review of pediatric diagnosis in patients with OGI between 2017 and 2020 at Henan Eye Hospital. Four hundred and one eyes of the patients younger than 17 years were included in this study. Open globe injury was classified according to the Birmingham Eye Trauma Terminology system (BETT). Age, sex, history, cause, month of trauma, treatment received, and outcomes were recorded. Visual acuity was documented according to standard visual acuity chart (decimals).

**Results:**

Four hundred and one eyes of patients were included in the study. The mean age was 6.6 ± 3.4 years with the range from 8 months to 16 years. Open globe injuries (OGIs) occurred most frequently in the 2–8 year age and significant male predominance was noted (70.8%). The incidence of pediatric OGIs was lowest in summer months while it increased in the winter months. The most common type of pediatric OGI was penetration (89%). Scissors/knife accounted for 22%, followed by pen/pencil (15.2%), and wood/bamboo sticks (14.5%) of all the pediatric OGIs. Among the injuries, the most frequently involved is the zone I (76.1%). Initially, 70.8% of the eyes received primary debridement and wound closure without any additional intervention, while only one eye has no possibility of anatomical reconstruction when it received an evisceration. After the initial management, 198 eyes received subsequent operation, including 44 eyes that underwent cataract removal + intraocular lens (IOL) implantation, and 24 eyes underwent IOL implantation. Finally, over 6 months of follow-up, 129 eyes (32.2%) got visual acuity (VA) of 0.3–1.5 and, 63 eyes (15.7%) got VA of 0.01–0.25, while 11 eyes (2.7%) were eviscerated.

**Conclusion:**

This study showed that pediatric OGIs in central China are most seen in 2–8-year age group with significant male predominance. Scissors/knife, pen/pencil, and wood/bamboo sticks accounted for over half of all cases. Pediatric OGIs often result in significant vision loss. In some severe cases (2.7%), evisceration was ultimately performed. We should call on the public to pay more attention to their children and build a safer environment for them.

## Introduction

Open globe injury (OGI) refers to the ocular trauma with full thickness wound of the eyewall (1). It is a major cause of non-congenital monocular visual loss in the pediatric population (2–4) and it can cause a lifelong impact on children. In spite of medical and technical advancements, pediatric OGIs may result in substantial visual morbidity and lifelong sequelae. Severe pediatric OGIs also impose financial burdens on society and families. In addition to the primary impact by the trauma itself, OGIs can result to amblyopia in younger children. Children are the most prone to getting their eyes hurt because of lesser ability to recognize hazards and less focused moves.

Henan province is a major agricultural province that lies in central China. Latest population census shows that the population of Henan was about 100 million (99,366 thousands) and the population of 0–15 years old was about 24,406 thousands. Henan Eye Hospital is one of the largest ophthalmic centers in Henan Province and receives large numbers of patients with pediatric ocular trauma every year.

With regards to ocular trauma, prevention is more important than treatment. In order to develop ocular safety education and injury prevention programs, it is important to understand the epidemiology and characteristics of OGI in pediatric patients. The aim of this study was to investigate the characteristics, treatments, and outcomes of pediatric OGIs diagnosed and treated at Henan Eye Hospital.

## Methods

We retrospectively reviewed all the patients who were admitted and treated at Henan Eye Hospital between 2017 and 2020. Most self-sealed globe injuries with low risk of infection will be treated by eye drops (antibiotics and others) and will not be admitted to hospital. However, self-sealed globe injuries but with high risk of infection and severe open globe injuries will be admitted to hospital for surgery or observation. Attending doctors will deal with simple OGI such as wound closure, ocular injection, and so on. Senior surgeon on duty will perform for complex surgery such as vitrectomy + IOFB removal, and so on. Patients will be referred to Ocular Trauma center if necessary after the primary treatment.

Four hundred and one eyes of 401 patients younger than 17 years were included in the study, with the cut-off age of 16, and were consistent with prior studies (5–7). The OGIs were classified according to the Birmingham Eye Trauma Terminology system (BETT) (1). This classification consists of rupture (full-thickness wound of the eyewall, caused by a blunt object), penetrating injury (entrance wound/s only, caused by sharp object/s), intraocular foreign body (IOFB), or perforating injury (separate entrance and exit wounds) (8). The zone of injury was defined as zone I (wound involves only cornea), zone II (wound extends into anterior 5 mm of sclera), and zone III (wound involves sclera extending more than 5 mm from the limbus) (8).

Visual acuity was documented according to the standard visual acuity chart (decimals).

Data were analyzed using Microsoft Office Excel 2007 (Microsoft, USA). Continuous and categorical variables were displayed as means ± standard deviation (SD) and percentages, respectively.

Ethics approval for the study was granted by Henan Eye Institute, Henan Eye Hospital, Henan Provincial People's Hospital Human Research Ethics Committee. The study adhered to the tenets of the Declaration of Helsinki.

## Results

**Figure 1:**
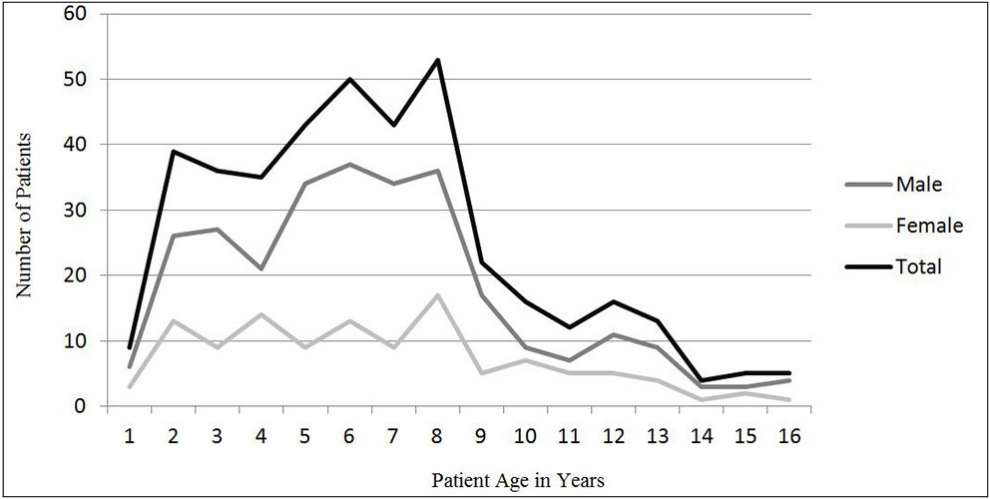
Sex and age distribution of OGI in children.

**Figure 2:**
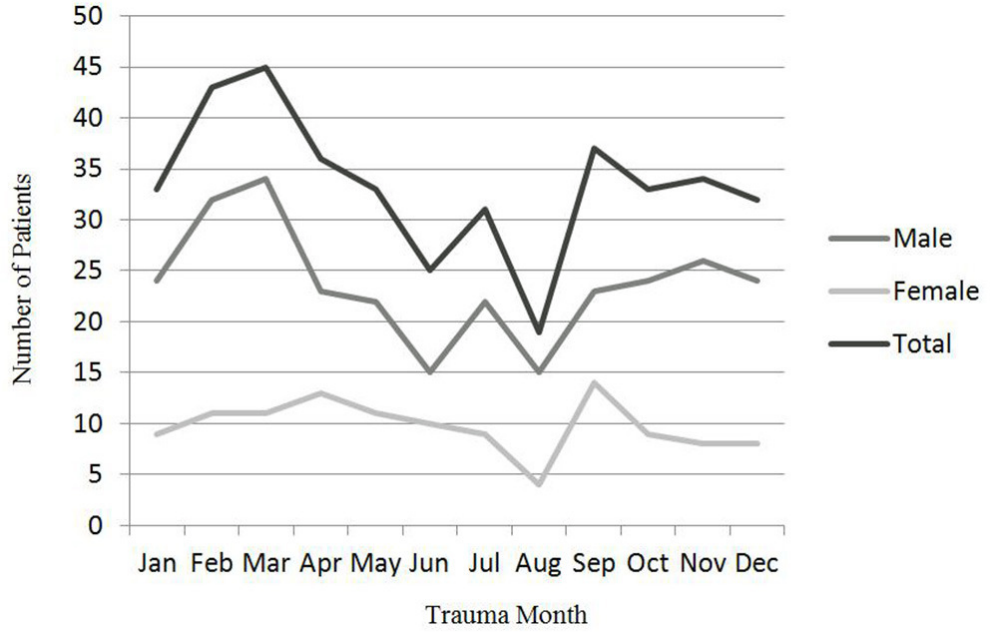
Sex and trauma month distribution of OGI in children.

In the 4 years between 2017 and 2020, 1,902 cases (1,908 eyes) of patients with OGI were admitted and treated at Henan Eye Hospital. Of all the OGIs, 401 eyes (21.0%) of 401 patients younger than 17 years were included in our analyses. The mean age was 6.6 ± 3.4 years with the range from 8 months to 16 years. The right eye was injured in 218 (54.4%) cases. Most frequently, OGIs occur in the 2–8 years age (Figure 1) and these patients aged 2–8 years accounted for 74.6% (299 patients) of all cases. Significant male predominance was noted (70.8%). Male-female ratio was 2.43. The incidence of pediatric OGI was low in summer months and was lowest in August (Figure 2).

Causative objects of the OGIs are listed in Table 1. Injuries were most often caused by scissors and knife (22.0%). Sixty-seven eyes (16.7%) were injured by scissors. Pen and pencil accounted for 15.2% cases (88 eyes) of pediatric OGIs.

**Table 1:** Causes of pediatric open globe injuries.

**Cause**	***n***	**%**
Scissors, knife	88	22.0
Pen, pencil	61	15.2
Wood or bamboo stick	58	14.5
Metal wire, metal stick	32	8.0
Fall, tumble	31	7.7
Toy	24	6.0
Glass	23	5.7
Fireworks, firecrackers	17	4.2
Needle, syringe	6	1.5
Flying stone	6	1.5
Key	3	0.8
Traffic accident	3	0.8
Lighter explosion	3	0.8
Straw	3	0.8
Door	3	0.8
Book	2	0.5
Beak of cock	2	0.5
Violence	2	0.5
Others^*^ and unknown	34	8.2
Total	401	100.0

* *Others include unusual causes such as fitness equipment, fishing line, and so on*.

**Figure 3:**
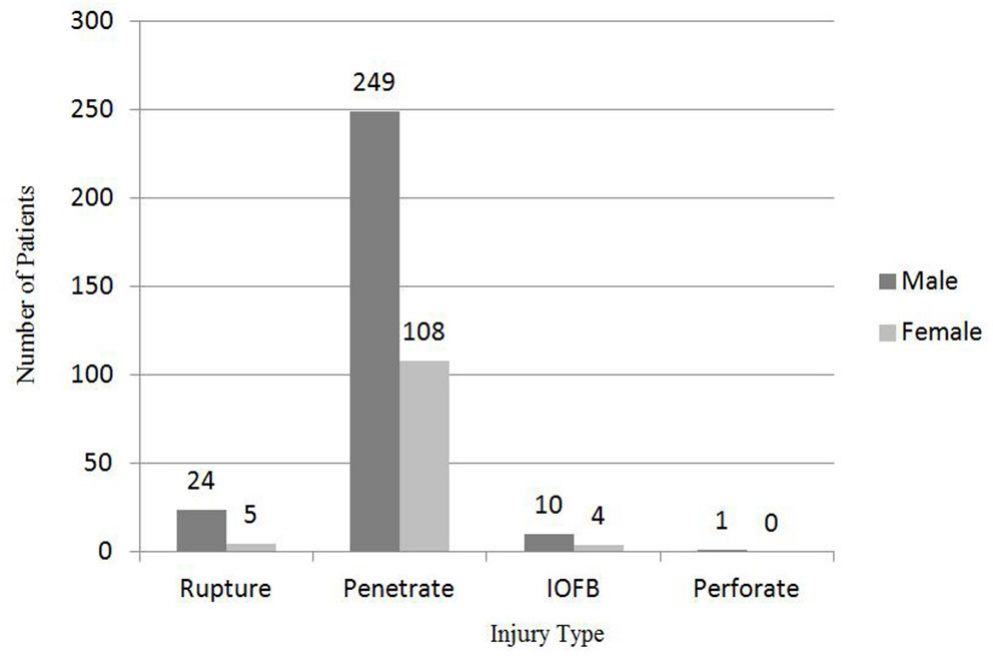
Sex and injury type distribution of OGI in children.

In the present study, the most common type of pediatric OGI was penetration (89%) (Figure 3). There was only one case of perforation in the present study. It was a 2-year old boy whose right eye was injured by a wood stick. Perforation was diagnosed during the secondary surgery (cataract removal +vitrectomy + silicone oil) 40 days after the wound closure. Fortunately, silicone oil was safely removed 3 months after the second surgery. Of all the 11 intraocular foreign body (IOFB) cases, IOFB was eyelash in 7 eyes.

**Figure 4:**
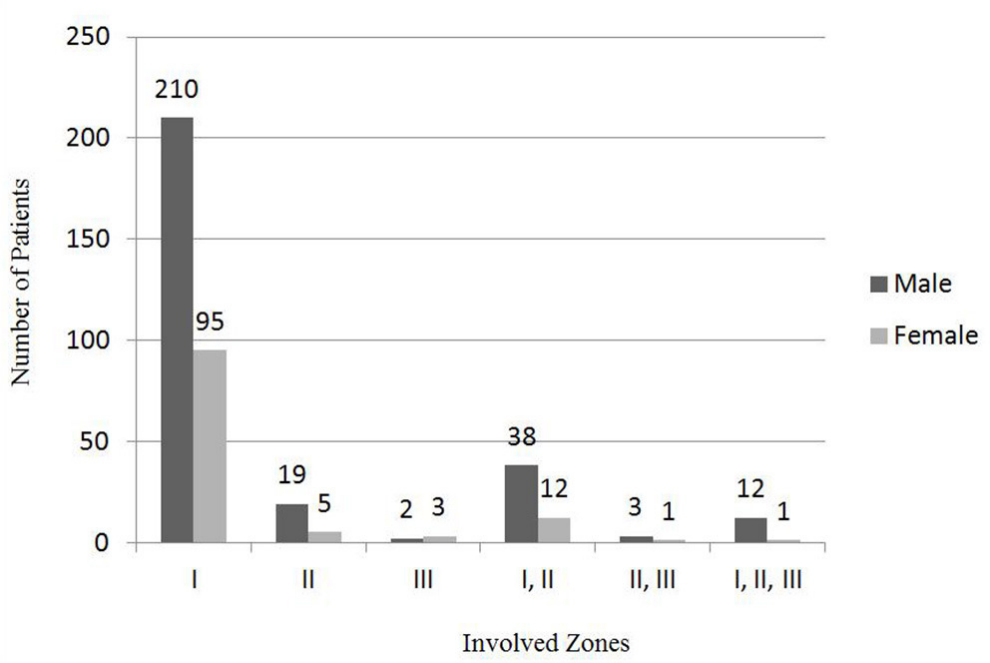
Distribution of gender and zones involved in pediatric OGI.

Injuries most frequently involved zone I (76.1%; *n* = 305) (I group) (Figure 4). Fifty pediatric OGIs (12.5%) involved zones I and II (I, II groups), and 24 eyes (12.5%) involved zone II (II group). Thirteen eyes (3.2%) involved zones I, II, and III (I, II, III group). Five eyes (1.2%) involved zone III (III group) and 4 eyes (1%) involved zones II and III (II, III group).

Initially, 70.8% eyes received primary debridement and wound closure without additional intervention, and only one eye has no possibility of anatomical reconstruction received evisceration (Table 2). Twenty-nine eyes (7.2%) received wound closure and cataract removal, while 16 eyes received wound closure and intraocular injection (antibiotics). Nine eyes (2.2%) received wound closure, cataract removal, and anterior vitrectomy. Seven eyes (1.8%) only received intraocular injection (antibiotics) because the injuries were self-sealed, and the infection was under control. Comprehensive management, at least, included vitrectomy, silicone oil tamponade, and other management such as wound closure, cataract removal, intraocular injection, and IOFB removal. Six eyes (1.5%) received comprehensive management as the primary surgery.

**Table 2:** Primary surgery.

**Surgical options**	***n***	**%**
Wound closure	284	70.8
Wound closure + cataract removal	29	7.2
Wound closure + intraocular injection	16	4.0
Wound closure + cataract removal + anterior vitrectomy	9	2.2
Wound closure + cataract removal + IOL implantation	2	0.5
Wound closure + cataract removal + IOFB removal	2	0.5
Wound closure + IOFB removal	2	0.5
Wound closure + cataract removal+IOFB removal	2	0.5
Wound closure + intraocular injection + IOFB removal + intraocular injection	1	0.25
Wound closure + cataract removal + anterior vitrectomy + IOFB removal+intraocular injection	2	0.5
Wound closure + anterior vitrectomy	1	0.25
Cataract removal + IOL implantation	3	0.75
Cataract removal + anterior vitrectomy + IOL implantation	3	0.75
Comprehensive management	6	1.5
Intraocular injection	7	1.8
Evisceration	1	0.25
Re-formation of anterior chamber	1	0.25
No surgery	30	7.5
Total	401	100

*IOL, intraocular lens; IOFB, intraocular foreign body*.

After the initial management, 198 eyes (49.4%) received subsequent operation (Table 3), including 44 eyes (22.2%) that underwent cataract removal + IOL implantation, while 24 eyes (12.1%) underwent IOL implantation. Twenty-two eyes (11.1%) received vitrectomy and silicone oil tamponade, and 21 eyes (10.6%) received cataract removal, vitrectomy, and silicone oil tamponade at the secondary intervention. Seven eyes got eviscerated at the secondary intervention. Another three eyes were eviscerated at the third, fourth, and fifth intervention, respectively. Eleven eyes were eviscerated eventually (including one eye that got eviscerated at the first intervention).

**Table 3:** Secondary intervention.

**Surgical options**	***n***	**%**
Cataract removal + IOL implantation	44	22.2
IOL implantation	24	12.1
Vitrectomy + silicone oil	22	11.1
Cataract removal + vitrectomy+silicone oil	21	10.6
Cataract removal	15	7.6
Cataract removal + anterior vitrectomy+IOL	14	7.1
Cataract removal + vitrectomy	12	6.1
Cataract removal + anterior vitrectomy	9	4.6
Intraocular injection	6	3.0
IOL implantation + anterior vitrectomy	5	2.5
IOL implantation + silicone oil removal	3	1.5
Anterior vitrectomy	3	1.5
Vitrectomy + IOFB removal+silicone oil	2	1.0
Cataract removal + vitrectomy + IOFB removal + intraocular injection	1	0.5
Cataract removal + vitrectomy + intraocular injection	1	0.5
Vitrectomy + intraocular injection	1	0.5
Evisceration	7	3.5
Others^*^	8	4.1
Total	198	100

* *Others include unusual intervention such treatment of iris cysts, iris and ciliary body surgery*.

*IOL, intraocular lens; IOFB, intraocular foreign body*.

Finally, over 6 months of follow-up, 129 eyes (32.2%) got visual acuity (VA) of 0.3–1.5, and 63 eyes (15.7%) got VA of 0.01–0.25, while 11 eyes (2.7%) were eviscerated (Table 4).

**Table 4:** Final outcomes of 401 eyes of pediatric OGIs.

**Outcomes**	***n***	**%**
0.3–1.5	129	32.2
0.01–0.25	63	15.7
HM-CF	8	2.0
Silicone oil dependence	30	7.5
Atrophy	5	1.3
Evisceration	11	2.7
Loss of follow-up	57	14.2
Others^*^	98	24.4
Total	401	100

* *Others include patients who was too young to test vision acuity*.

*HM, hand movement; CF, counting fingure*.

**Figure 5:**
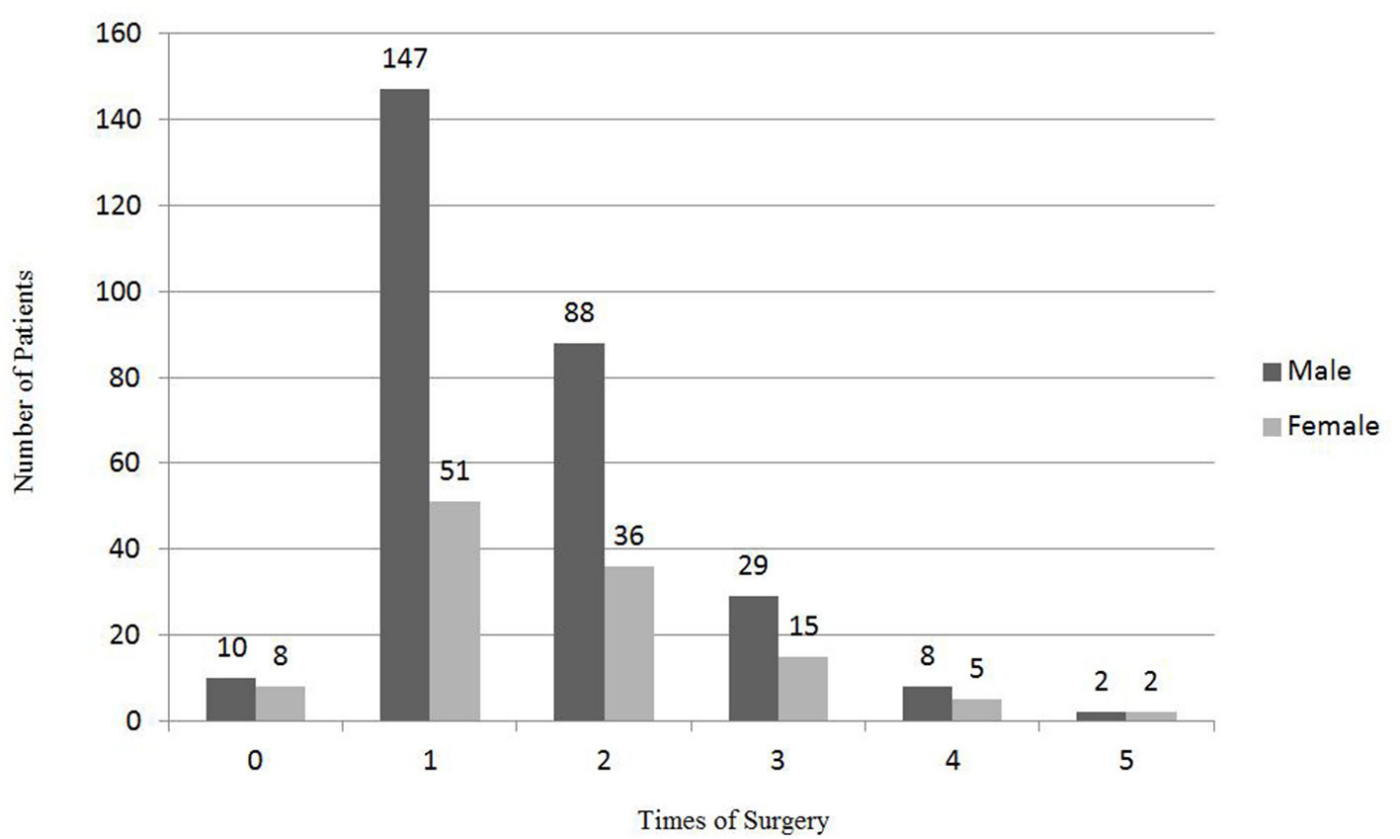
Distribution of gender and times of surgery for pediatric OGI.

Of all the 401 pediatric OGI cases, 18 eyes (4.5%) had never received any surgery because they were self-sealed globe injuries. Almost half (49.4%; *n* = 198) of the eyes sampling received only once surgery (Figure 5). One hundred and twenty-four eyes (30.9%) underwent the surgeries twice and 44 eyes (11.0%) underwent the surgeries for three times. Thirteen (3.2%) and four eyes (1%) received four times and five times of surgeries, respectively.

In this study, 36 eyes (9%) were diagnosed with endophthalmitis. One case developed endophthalmitis 2 years after the ocular trauma by scissors due to the reopening and the leakage of the primary limbus wound. Twenty eyes (5%) were diagnosed with endophthalmitis at first management and received an intraocular injection (antibiotics) or comprehensive management. Three eyes were eviscerated at the second, third, and fifth intervention, respectively. One eye got atrophic after six interventions. One eye of a 2-year old boy got atrophic after the primary wound closure without further ocular treatment because of high fever and poor general condition. The infection was controlled at pediatric department. Ten eyes received intraocular injection (antibiotics) and/or comprehensive management. One eye achieved VA of 0.8 at the end of follow-up.

Two eyes (0.5%) developed iris cyst and 1 eye (0.25%) developed sympathetic ophthalmia 3 weeks after the ocular trauma.

## Discussion

The Birmingham Eye Trauma Terminology system (BETT) provides an unambiguous, consistent, simple, and comprehensive system to describe any types of mechanical globe trauma. According to BETT, open-globe injury (OGI) refers to the ocular trauma with full thickness wound of the eyewall (1) and it consists of a rupture, penetrating and perforating IOFB.

In this study, 21% of all OGIs were children younger than 17 years old. The proportion of pediatric OGI was 9.26–22.99% in other previous studies from other countries (9–12). However, in the study of Batur et al. (13), the proportion was 67.7%. Similarly, it was 43.1–68.9% in other studies conducted in Turkey (14, 15). The high proportion of pediatric OGI in children could be attributed to the high population growth rate and high proportion of the children.

The male-female ratio was 2.43 in our study. The male-female ratio was 1.44–5.13 in other previous studies (11–13, 16–22). The differences of male-female ratio among different regions or countries could be related to many factors.

Compared to the study of Batur et al. (13), there were many similarities and other differences. Both studies have evaluated patients aged 16 years or younger. The mean age of the patients was 7 ± 3.7 years in the study of Batur et al. (13), and it was 6.6 ± 3.4 years in our study. According to the study of Batur et al. (13), OGI occurred most frequently in the 3-to-7-year age group, and it was 2-to-8-year age group in our study. Except the difference of proportion of OGIs in children, another interesting difference was the month of distribution of OGI in children. The incidence of OGI decreased in winter months and increased in summer months in the study of Batur et al. (13), starting in May and reaching a peak in August. Conversely, our study showed that the incidence of pediatric OGI was low in summer months and it was lowest in August (4.7%; *n* = 19) (Figure 2). It was highest in March (11.2%; *n* = 45). The reason of the difference between the two studies was unclear. Temperature in Henan, China is similar to that in Sanliufa, Turkey and it is highest in August every year.

Causative objects of the ocular trauma in our study were scissors, knives, pen/pencil, sticks, and other sharp objects. It was consistent with other previous studies (11, 13, 17).

The primary repair to maintain the integrity of the globe is the appropriate choice for initial treatment of OGIs (13). Simple primary repair was performed in 70.8% of eyes in our study. In some complicated cases such as cataract with rupture of capsule, IOFB, vitreous prolapse, and suspect of endophthalmitis, wound closure combined with other procedures were carried out in 18% of eyes.

Of all the 401 pediatric OGI cases, 18 eyes (4.5%) with self-sealed globe injuries had never received any surgery and almost half (49.4%) of the eyes received one surgery. One hundred and twenty-four eyes (30.9%) underwent surgeries for two times and 44 eyes (11.0%) underwent surgeries for three times. Thirteen (3.2%) and four eyes (1%) received surgeries for 4 times and 5 times, respectively. It was a little different from the result of Batur et al. (13). In the study of Batur et al. (10), 64.4% of the eyes required one surgical procedure, 26.1% required two, and 6.2% required three separate procedures.

One of the major differences between pediatric and adult OGIs is the difficulty of assessing VA in children immediately after the trauma and at the follow-up. It is more difficult to assess VA immediately after trauma in children because of pain, discomfort, and fear among them. Additionally, manipulation of the globe may cause further damage and expulsion of intraocular contents. Almost half of the patients could not provide VA assessment initially and 24.4 patients could not carry out VA assessment at follow-up. In the present study, 129 eyes (32.2%) got visual acuity (VA) of 0.3–1.5 and 63 eyes (15.7%) got VA of 0.01–0.25.

In this study, except one case developed endophthalmitis 2 years after the ocular trauma by scissors due to the reopening and leakage of the primary limbus wound, 35 eyes (8.7%) were diagnosed with endophthalmitis at primary or secondary management. In the study of Behbehani et al. (19), only one eye (1.1%) developed endophthalmitis of all the pediatric 95 patients. In the study of Lesniak et al. (8), there was no one who developed endophthalmitis of all the pediatric 89 patients. In another study, of all the 213 patients, eight (3.7%) eyes had the clinical diagnosis of posttraumatic endophthalmitis (16). While in the study of Choovuthayakorn et al. (12), endophthalmitis occurred in 15 of the 49 patients (30.6 %). The incidence of post-traumatic endophthalmitis is higher in the subjects living in rural areas (23).

This study reviewed the characteristics, managements, and outcomes of pediatric open globe injury (OGI) in central China. It has several potential limitations. First, this is a retrospective study in nature. Second, the data were collected in a single institution. Third, the differences in patient selection and surgical technique between experts could lead to bias. Thus, a prospective study is warranted in the future.

Nonetheless, to our knowledge, the present study is the first to demonstrate the characteristics, managements, and outcomes of pediatric open globe injury (OGI) in central China. It is helpful to carry out preventive program to reduce the occurrence of ocular trauma in children. The authors of this study usually published relevant publicity and education through WeChat, the website of the hospital and other We-Media. The authors had also been invited to the local TV show and broadcast programs every year to educate the public how to prevent ocular trauma in children. However, it seems far from enough. The parents or guardians should pay more attention to the children and build much safer environment for their growth. However, the left-behind children in rural areas do not usually get intensive care from their grandparents or other guardians. Fortunately, the government had realized the problem of these left-behind children. More and more of these children will stay with their parents.

## Data Availability Statement

The original contributions presented in the study are included in the article/supplementary material, further inquiries can be directed to the corresponding author/s.

## Ethics Statement

The studies involving human participants were reviewed and approved by Henan Eye Institute, Henan Eye Hospital, Henan Provincial People's Hospital Human Research Ethics Committee. Written informed consent to participate in this study was provided by the participants' legal guardian/next of kin.

## Author Contributions

XZ and HC organized the database. HC performed the statistical analysis and wrote the first draft of the manuscript. All authors contributed to conception, design of the study, manuscript revision, read, and approved the submitted version.

## Conflict of Interest

The authors declare that the research was conducted in the absence of any commercial or financial relationships that could be construed as a potential conflict of interest.

## Publisher's Note

All claims expressed in this article are solely those of the authors and do not necessarily represent those of their affiliated organizations, or those of the publisher, the editors and the reviewers. Any product that may be evaluated in this article, or claim that may be made by its manufacturer, is not guaranteed or endorsed by the publisher.
